# The Otoacoustic Emissions in the Universal Neonatal Hearing Screening in China and India: An Update on the Asian States (2005 to 2025)

**DOI:** 10.3390/children13060751

**Published:** 2026-05-28

**Authors:** Stavros Hatzopoulos, Ludovica Cardinali, Piotr Henryk Skarzynski, Giovanna Zimatore

**Affiliations:** 1 Clinic of Audiology & ENT, University of Ferrara, 44121 Ferrara, Italy; 2 Department of Life Science, Health, and Health Professions, Link Campus University, 00165 Rome, Italygiovanna.zimatore@uniecampus.it (G.Z.); 3 Heart Failure and Cardiac Rehabilitation Department, Faculty of Medicine and Dentistry, Medical University of Warsaw, 02-005 Warsaw, Poland; 4 Institute of Sensory Organs, 05-830 Nadarzyn, Poland; 5 World Hearing Center, Department of Teleaudiology and Screening, Institute of Physiology and Pathology of Hearing, 02-042 Warsaw, Poland; 6 Department of Theoretical and Applied Sciences Applied Physics, DiSTA, eCampus University, 22060 Novedrate, Italy

**Keywords:** Asia, congenital hearing loss, newborn hearing screening, otoacoustic emissions, well babies, NICU, bilateral hearing loss

## Abstract

**Highlights:**

**What are the main findings?**

- China has developed large-scale, system-integrated UNHS programs with high and stable coverage, whereas India shows predominantly hospital-based implementations with heterogeneous coverage and follow-up.
- OAE-based screening, particularly in combination with AABR, is effective in both settings, but loss to follow-up and variability in program structure remain major limitations.

**What is the implication of the main finding?**

- Strengthening follow-up systems and integrating screening, diagnosis, and rehabilitation into national health frameworks are essential to improve early detection outcomes.
- Standardized protocols and reporting, along with increased focus on long-term outcomes, are needed to enhance comparability and optimize neonatal hearing screening programs globally.

**Abstract:**

Background: China and India represent a large proportion of the Asian birth cohort and have produced extensive but heterogeneous evidence on neonatal hearing screening. This scoping review summarizes studies published between 2005 and 2025 on otoacoustic-emission-based neonatal hearing screening programs in these countries, with emphasis on program implementation, screening coverage, the prevalence of congenital and bilateral hearing loss, follow-up, and intervention pathways. Methods: Searches were conducted in PubMed, Scopus, and Google Scholar using predefined keywords. Studies reporting screening protocols, coverage, prevalence, or follow-up outcomes were included. The standard English language filter was used. A total of 19 papers were considered for this review. Results: The data from the two assessed Asian states show two clearly different screening implementation profiles. In China, universal hearing screening has evolved into a large-scale and increasingly standardized system, supported by technical specifications and regional or municipal databases. The reported screening coverage was 85.8% in early rural programs, 93.6% in Shanghai, and 97.9% in Liuzhou. National institutional surveys indicate that UNHS has now been substantially implemented in many regions. Reported hearing loss prevalence estimates generally ranged from 1.66 to 3.43 per 1000 newborns, although follow-up and regional equity remain problematic, especially in rural settings. In India, the evidence is dominated by tertiary-hospital feasibility studies rather than a uniformly implemented national program. Reported hearing loss prevalence estimates varied more widely, from 0.29 to 5.60 per 1000 screened newborns, largely reflecting differences in study design, screening timing, referral completion, and population risk profile. Across both countries, OAE-based two-stage or sequential OAE + AABR protocols reduced referral rates and improved case identification, but loss to follow-up remained a recurrent limitation. Conclusions: China and India provide complementary models of neonatal hearing screening expansion. China demonstrates the effects of system-level scale-up, whereas India highlights the feasibility and constraints of hospital-based implementation in a highly diverse healthcare environment. Future priorities include stronger follow-up systems, harmonized reporting standards, and broader dissemination of outcome data through peer-reviewed publications.

## 1. Introduction

Neonatal hearing screening (NHS) is a core component of early hearing detection and intervention, as timely identification of congenital hearing loss is strongly associated with improved speech, language, cognitive, and social outcomes [1,2]. Otoacoustic emissions (OAEs), used alone or in combination with automated auditory brainstem response (AABR), remain the most widely adopted first-line tools because they are rapid, non-invasive, and relatively economical [3,4,5]. The previous Asian scoping review from our group [6] deliberately excluded China and India because their scale, healthcare heterogeneity, and screening infrastructures would have disproportionately influenced the synthesis of the paper.

A dedicated review focused on China and India is justified for both demographic [7] and methodological reasons. China has progressively developed a large and increasingly standardized Universal NHS (UNHS) system, supported by provincial, municipal, and national analyses. India, in contrast, has produced an extensive body of hospital-based feasibility studies, but without a uniformly implemented nationwide UNHS framework [8]. Taken together, these two countries offer an opportunity to compare two distinct pathways of program development within Asia: system-level expansion in China and institution-driven implementation in India. The differences in the implementation of UNHS between China and India are also shaped by their distinct economic contexts. China’s sustained economic expansion has facilitated major investments in public health, enabling the progression from local pilot projects to coordinate nationwide programs. In contrast, although India is the world’s most populous nation and an emerging economic power, it still faces considerable disparities in healthcare infrastructure and resource distribution. This may partly account for the largely hospital-based and feasibility-focused character of the available evidence on hearing screening in the country. These economic and infrastructural disparities should therefore be considered when interpreting the comparative results of this review. Even though hearing screening is considered a required clinical practice, NHS programs seem to face many implementation obstacles, similar to what we have previously described in the study of the European [9] and African NHS realities [10,11].

As in the previous manuscripts on European [9], African [10], and Asian NHS data [6], the present scoping review addresses five key questions: (i) which regions/areas have implemented NHS-UNHS programs; (ii) what proportion of newborns is screened; (iii) which OAE-based protocols are most frequently employed; (iv) what the reported prevalences of congenital and bilateral hearing loss are; and (v) which risk factors and intervention pathways are described.

## 2. Materials and Methods

This scoping review was designed as the second Asian update in our series on OAE-based neonatal hearing screening and followed the PRISMA-ScR (Preferred Reporting Items for Systematic Reviews and Meta-Analyses extension for Scoping Reviews) guidelines (the check list is reported in Supplementary Materials). In contrast to the previous Asian review, which excluded China and India, the present study focuses exclusively on these two countries. The review period was set from January 2005 to December 2025 in order to capture both the early implementation phase and the more recent consolidation of screening practices. The structure follows the same general approach used in our earlier papers on the European, African, and Asian data.

Searches were conducted in PubMed, Scopus, and Google Scholar using combinations of the following MESH terms: “otoacoustic emissions”, “OAE”, “TEOAE”, “DPOAE”, “automated auditory brainstem response”, “newborn hearing screening”, “universal neonatal hearing screening”, “congenital hearing loss”, “China”, and “India”. Studies were eligible when they reported original neonatal or infant hearing-screening data, described program implementation or coverage, or analyzed prevalence, referral, follow-up, or intervention outcomes. Narrative commentaries and papers not reporting primary data were excluded from the final synthesis. Review papers were used for contextual interpretation when relevant. Baseline data were provided by the extensive survey-based study by Neumann et al. [12] in 2020, which examined healthcare systems in 196 states globally. For additional information, see Table A1 in Appendix A.

Preference was given to peer-reviewed studies with transparent methods, clearly reported screening protocols, and extractable data on sample size, screening pathway, or outcome metrics. When multiple reports from the same program were available, the most informative or recent paper was prioritized, while landmark earlier studies were retained when necessary to document program evolution. Because the Chinese and Indian literature is extensive and highly heterogeneous, the review was intentionally scoped rather than designed as a meta-analysis.

The inclusion criteria for the current review were established as follows: (i) the geographical affiliation of the study (China and India), (ii) the size of the screened population, with preference given to larger cohorts, and (iii) the recency of data, selecting the most up-to-date study or studies available for each country.

**Figure 1:**
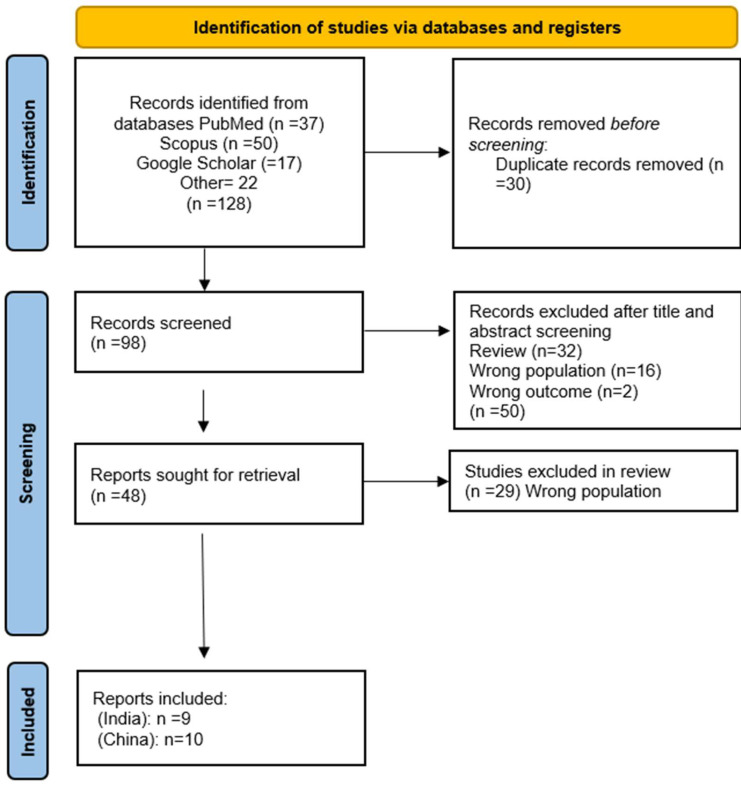
Flow diagram of literature search, according to PRISMA criteria https://www.prisma-statement.org/prisma-2020, (accessed 15 December 2025), with the steps followed in the manuscript selection procedure. After the application of the selection criteria the initial 128 manuscripts were reduced to 19 [12,13,14,15,16,17,18,19,20,21,22,23,24,25,26,27,28,29,30].

Two independent reviewers assessed the retrieved material, resulting in a final selection of 48 eligible papers. At the title and abstract screening stage, articles clearly involving non-neonatal populations (e.g., adults or school-age children) were excluded without full-text retrieval. A second exclusion stage, conducted during full-text review, identified an additional 29 papers that had appeared potentially eligible at the abstract level but were found upon full-text assessment to involve predominantly school-age children rather than neonates or infants; these were therefore excluded, resulting in a final selection of 19 manuscripts, 10 for China and 9 for India. In studies employing two-stage OAE + AABR protocols, a pass was defined as passing the first-stage OAE; infants failing OAE were referred for AABR. A final fail, and thus referral for diagnostic evaluation, was assigned only when both OAE and AABR were not passed. In studies combining OAE with genetic or metabolic screening, the audiological pass/fail criterion was based solely on OAE results, while a positive genetic or metabolic finding was treated as an indicator for enhanced surveillance rather than an immediate fail outcome. The PRISMA flow diagram is presented in Figure 1, while the studies included in the review are summarized in Table 1.

## 3. Results

Table 2, at the end of this section, provides a summary of the data that were retrieved from the 19 chosen studies. Table 3 shows the differences in the NHS practices between the two assessed Asian states.

The analytical data from each study (grouped per country) are presented below:

**Table 2:** Data presenting the NHS activities in China and India. The data are presented in alphabetical order per state. Abbreviations: OAE: Otoacoustic Emissions; TEOAE: Transient Evoked OAE; DPOAE: Distortion Product OAE; AABR: Automated Auditory Brainstem Response; ABR: Auditory Brainstem Response; PCHL: Permanent Childhood HL; UNHS: Universal Newborn Hearing Screening, NR: Not Reported Information.

n	State	Region/City or Town	Screening Protocol (OAE/ABR)	Hearing Loss Prevalence	Causes/Risk Factors	Author (First)	Year
1	China	Multicenter	OAE + genetics	1.9/1000	Hereditary	Wang	2011
2	China	Multicenter	OAE vs OAE + AABR	3.0/1000	Model	Huang	2012
3	China	Tianjin	OAE + genetics	2.3/1000	Pathogenic variants in GJB2, SLC26A4, mtDNA	Zhang	2013
4	China	Multicenter	OAE + genetics	NR	GJB2 mutations; non-syndromic HL	Dai	2015
5	China	Southern China	OAE + genetics	2.7/1000	GJB2 and mitochondrial mutations	Peng	2016
6	China	Shandong	Automated OAE	NR	gestational diabetes mellitus (GDM)	Zhou	2021
7	China	Beijing	UNHS referral	NR	Referral outcomes	Li	2023
8	China	Hospital-based	OAE + metabolic	NR	Metabolic disorders (e.g., hyperbilirubinemia)	Ren	2025
9	China	Liuzhou (Guangxi)	DPOAE + AABR (two-step UNHS)	2.25/1000 (PCHL); 0.33% total HL	Metabolic disorders (e.g., hyperbilirubinemia)	Wu	2017
10	China	Shanghai	OAE + AABR	1.66/1000	Large-scale program; integrated screening–intervention–rehabilitation system; high coverage (93.6%)	Chen	2017
11	India	Vellore	DPOAE (2-stage) + AABR confirm DPOAE + ABR	6.0/1000	NICU	John	2009
12	India	Chandigarh	TEOAE + ABR	NR	Timing	Bansal	2008
13	India	Jabalpur	DPOAE + ABR	8.9/1000	High-risk	Sachdeva	2017
14	India	Ballabgarh	DPOAE (2-stage) + ABR confirmation	5.0/1000	CMV	Dar	2017
15	India	Pune	OAE + ABR	3.54/1000	Low birth weight, hyperbilirubinemia, craniofacial anomalies	Parab	2018
16	India	Lucknow	OAE + ABR	7.0/1000	Repeat OAE reduced referrals; program implementation feasibility	Upadhyay	2022
17	India	Panchkula	OAE → ABR	8.2/1000	Prematurity	Rawat	2023
18	India	Pune	OAE + ABR (two-step UNHS)	5.41/1000 (overall); 9.11/1000 high-risk; 1.49/1000 well-baby	NICU stay, low birth weight, IUGR, RDS, hyperbilirubinemia; high loss to follow-up	Kapadia	2022
19	India	Odisha (policy/program setting),	Decision-tree model: OAE vs portable AABR	NR (economic model; not an observed population prevalence)	Resource constraints; device portability; at-risk prevalence cited from literature	Sahoo	2024

**Table 3:** Summary comparison between China and India.

Domain	China	India
Implementation	Large-scale UNHS	Mostly hospital-based
Coverage	High (>85–95%)	Variable
Prevalence range	1.9–3.0/1000	3–9/1000
Protocols	OAE + AABR + genetics	OAE + ABR
Main limitation	Regional inequality	Follow-up & infrastructure

### 3.1. China

Wang et al. [13] examined the clinical utility of concurrent newborn hearing and genetic screening in a cohort of 14,913 Chinese newborns. All infants underwent OAE-based hearing screening combined with genetic testing for common deafness-associated mutations. The study reported a hearing loss prevalence of approximately 1.9 per 1000 live births. Importantly, genetic screening identified a significant proportion of infants at risk for delayed-onset or progressive hearing loss who would not have been detected by physiologic screening alone. The authors demonstrated that concurrent gene screening improves continuity of care by enabling early surveillance and targeted follow-up.

A multicenter economic evaluation simulated all neonates born between 2007 and 2009 across eight provinces in China (including Beijing, Shandong, Guangdong, and Zhejiang). Universal and targeted UNHS strategies using OAE followed by AABR were compared. Although the study by Huang et al. [14], focused on cost-effectiveness, congenital sensorineural hearing loss prevalence was assumed to be approximately 1–3 per 1000 live births based on provincial databases. The study supports universal UNHS in economically developed provinces. The analysis demonstrated that universal newborn hearing screening is cost-effective in economically developed regions of China, particularly when combined with two-stage OAE and AABR protocols, providing strong policy-level support for nationwide UNHS implementation.

Zhang et al. [15] reported the implementation of a large-scale newborn hearing screening program combined with concurrent genetic screening in 58,397 neonates in Tianjin, China. All newborns underwent otoacoustic emission (OAE) screening shortly after birth, alongside genetic testing targeting GJB2, SLC26A4, and mitochondrial variants. The overall prevalence of confirmed hearing loss was approximately 2.3 per 1000 live births. The combined screening approach enabled identification of infants at risk for delayed-onset or progressive hearing loss, highlighting the added value of genetic testing within UNHS programs.

Dai et al. [16] conducted a genotype–phenotype correlation study in patients with non-syndromic hearing loss across multiple centers in China. Although not a population-based newborn screening study, the work provided important insights into the molecular epidemiology of hearing loss, focusing on GJB2 mutations. The study demonstrated substantial variability in severity, laterality, and progression of hearing loss, reinforcing the relevance of genetic screening as a complement to physiologic UNHS methods.

Peng et al. [17] evaluated a combined physiologic and genetic newborn hearing screening strategy in 9317 newborns across multiple centers in Southern China. Standard OAE screening was supplemented with targeted genetic testing, primarily for GJB2 and mitochondrial mutations. The prevalence of confirmed congenital hearing loss was approximately 2.7 per 1000 live births. The study confirmed that genetic screening improves early risk identification and follow-up planning, particularly in infants who pass initial OAE screening.

Zhou et al. [18] investigated the association between gestational diabetes mellitus (GDM) and neonatal hearing screening outcomes in 666 newborns in Weifang, Shandong Province. OAE-based screening revealed significantly higher abnormal results among infants born to mothers with GDM compared with controls, although the confirmatory prevalence of permanent hearing loss was not reported. The findings highlight maternal metabolic disorders as important modifiers of neonatal auditory outcomes and the need for targeted follow-up.

Li et al. [19] analyzed long-term audiological outcomes in 1839 children referred from a universal newborn hearing screening program in Beijing over a nine-year period. Screening protocols included OAE and AABR followed by comprehensive diagnostic assessment. More than half of the referred children were diagnosed with hearing impairment, most commonly bilateral sensorineural hearing loss. The study underscored the importance of structured referral systems and long-term follow-up within large urban UNHS programs.

Ren et al. [20] explored the association between neonatal hearing screening outcomes and common metabolic disorders in a hospital-based cohort in China. Using routine OAE-based UNHS data correlated with neonatal metabolic screening results, the study identified significant associations between abnormal hearing screening outcomes and conditions such as hyperbilirubinemia and thyroid dysfunction. Although population prevalence per 1000 live births was not reported, the findings suggest that metabolic abnormalities may influence early auditory function.

Wu et al. [21] reported a two-step UNHS program (DPOAE + AABR) in 19,098 newborns, achieving high screening coverage (~98%) but low follow-up compliance (~42%). The prevalence of permanent congenital hearing loss was 2.25/1000, with higher rates in NICU infants. Significant rural–urban disparities were observed, particularly in access to diagnostic services.

Chen et al. [22] reported a large-scale UNHS program in Shanghai involving over 1.5 million newborns, achieving high screening coverage (93.6%). The prevalence of congenital hearing loss was 1.66/1000, consistent with international estimates. The study demonstrated the feasibility and cost-effectiveness of an integrated screening system, although further improvements were needed in follow-up and rehabilitation services.

### 3.2. India

John et al. [23] conducted one of the earliest pilot universal newborn hearing screening studies in India, screening 500 neonates at a tertiary care center in Vellore between 2006 and 2007. A two-stage protocol using automated otoacoustic emissions followed by automated auditory brainstem response testing was employed. The study demonstrated the feasibility of integrating UNHS into routine postnatal care and provided early evidence of congenital hearing loss det ection in the Indian hospital setting.

The protocol-based study by Bansal et al., 2008, [24] screened 2659 infants aged 0–3 months in Chandigarh, India, using TEOAE with repeat testing and confirmatory ABR. After follow-up, permanent hearing loss prevalence was approximately 0.3–0.5%. The authors proposed delayed screening at immunization visits to reduce false positives in developing countries.

Sachdeva et al. [25] reported outcomes from a hospital-based newborn hearing screening program conducted in 2254 neonates between July 2015 and May 2016 in Jabalpur. Screening employed distortion product OAE with confirmatory brainstem evoked response audiometry. The study documented a relatively high prevalence of hearing impairment among high-risk neonates, reinforcing the need for structured UNHS programs in central India.

Dar et al. [26] screened 1720 newborns in a prospective rural study conducted in Ballabgarh, Haryana, India. Screening involved DPOAE at birth, repeat OAE at 2–4 weeks, and confirmatory ABR. Permanent congenital hearing loss prevalence was 0.64%, with a markedly higher rate among CMV-positive infants (10%). The study highlights the contribution of congenital CMV to bilateral SNHL in low-resource rural settings.

Parab et al. [27] screened 8192 neonates in rural Maharashtra between 2014 and 2016 using OAE followed by ABR confirmation. The study reported higher hearing loss prevalence in high-risk infants compared with well babies and demonstrated the feasibility of large-scale screening in rural communities. These findings highlighted persistent rural–urban disparities in hearing health services.

Upadhyay et al. [28] reported prospective outcomes of a UNHS program involving 2676 neonates screened between 2018 and 2020 at a tertiary care center in Lucknow. A two-stage OAE-based protocol with diagnostic confirmation demonstrated high screening coverage, acceptable follow-up rates, and prevalence estimates comparable to other Indian studies, supporting scalability in urban tertiary hospitals.

Rawat et al. [29] conducted a pilot UNHS study in 506 neonates between 2021 and 2022 in Panchkula, North India, using a two-stage TEOAE protocol followed by ABR. The study observed higher referral and confirmed hearing loss rates among high-risk infants and highlighted the importance of systematic follow-up to minimize loss to follow-up in secondary care settings.

Kapadia et al. [8] reported a decade-long UNHS program (OAE followed by ABR) in 5542 neonates at a tertiary care center in India, including both well-baby and high-risk populations. The overall incidence of hearing loss was 5.41/1000, with higher rates in high-risk infants (9.11/1000) compared to well babies (1.49/1000). The referral rate after initial OAE screening was 11.72%, while follow-up compliance for confirmatory ABR was limited. Major risk factors included NICU stay, low birth weight, intrauterine growth restriction, respiratory distress syndrome, and hyperbilirubinemia.

Sahoo et al. [30] performed a cost-effectiveness analysis of universal newborn hearing screening strategies in India, comparing portable automated ABR with conventional OAE-based approaches. Using national epidemiological estimates, the study demonstrated that portable ABR may improve true case detection and be economically viable in resource-limited settings, particularly when deployed at scale within public health systems.

A comparison of China and India is reported in Table 3. 

## 4. Discussion

In our earlier assessments of NHS practices in Europe [9], Africa [10,11], and Asia (apart from China and India [6]), we highlighted and addressed the grave consequences of a delayed detection of hearing impairment in the language development of the infant population. The two scoping reviews’ goals were to assess the NHS data that is currently available in the Asian states and any potential updates since Neumann et al.’s 2020 estimations [12]. Eight of the sixteen studies used in this study were published after 2020. In order to address the five issues posed in both the preceding article and the Section 1, the reported data were combined in the same five major areas.

(i) States Implementing NHS-UNHS Programs

China and India illustrate two different models of neonatal hearing-screening development. In China, UNHS has progressed from local and rural pilot programs to large municipal databases and multi-institutional national surveys, indicating that organized implementation has been achieved across many regions, although with marked east–midland–west inequalities. India, by contrast, remains characterized largely by tertiary-care and city-based initiatives, with strong evidence of feasibility but no equally uniform nationwide implementation framework. This difference is methodologically important because China contributes system-level policy and coverage data, whereas India contributes predominantly institution-level implementation evidence.

(ii) Proportion of Newborns Screened

The proportion of screened newborns was generally higher and more consistently reported in China than in India. Chinese studies document 85.8% screening in early rural multi-county programs, 93.6% coverage in Shanghai over a decade-long database, and 97.94% capture in Liuzhou. By contrast, Indian studies usually report single-center experience, with some large successful hospital programs but also marked attrition at program entry and follow-up; in one tertiary academic study, only 773 of 2323 admitted babies underwent the first OAE. These findings suggest that coverage in China is increasingly embedded at the health-system level, whereas in India it remains highly dependent on local infrastructure, staffing, and parental compliance.

(iii) Screening Protocols and OAE Technologies

Both countries relied heavily on OAE-based screening, but China more often documented standardized sequential or combined protocols, while India more often reported pragmatic tertiary-hospital workflows. In China, TEOAE or DPOAE was commonly used as the first-line tool, with AABR or diagnostic ABR incorporated for NICU infants, second-stage assessment, or confirmatory diagnosis. Chinese studies also showed that adding AABR after failed TEOAE reduced referral rates without increasing misdiagnosis. In India, most studies used TEOAE or DPOAE for first screening and BERA/ABR for confirmation, often in two-tier or multi-stage protocols. The Indian literature repeatedly showed that OAE-only early screening is feasible and economical, but generates a higher false-positive burden when testing occurs too early or when follow-up systems are weak. Thus, the comparative evidence supports OAE-based screening as the operational backbone in both countries, but suggests that protocol optimization depends chiefly on timing, confirmatory ABR access, and follow-up retention. Referral rates were inconsistently reported across the included studies. Where available, rates ranged from approximately 5% to over 11% after initial OAE screening (e.g., 11.72% in Kapadia et al. [8]), depending on the screening protocol, timing of testing, and population risk profile (well-baby vs. NICU). This variability further underscores the need for standardized reporting in future UNHS programs.

(iv) Prevalence of Congenital and Bilateral HL

Reported prevalence estimates were variable in both countries, but the Chinese data were generally clustered within internationally plausible ranges when derived from large cohorts. Shanghai reported a prevalence of 1.66 per 1000, Liuzhou reported permanent hearing loss at 2.25 per 1000, and the cohort estimated congenital hearing loss at 3.43 per 1000. In India, prevalence estimates were more heterogeneous, ranging from 0.29% in a recent tertiary-hospital study to 9.11 per 1000 in an earlier weighted estimate from a standardized tertiary-care population. The variability in India likely reflects smaller cohorts, differing definitions of outcome, variable rescreen completion, and the frequent mixing of well-baby and high-risk Neonatal Intensive Care Unit (NICU) populations. Bilateral hearing-loss estimates are more consistently reported in recent Indian studies, including 2 per 1000 in the Raipur cohort, but remain less systematically documented than in the largest Chinese datasets.

(v) Causes Leading to HL and Intervention Strategies

Across both countries, the principal risk factors were broadly consistent with international literature, but the Chinese evidence was more systematically quantified. In China, congenital hearing loss was associated with craniofacial anomalies, NICU admission, family history, advanced maternal age, and, for sensorineural loss, exchange transfusion and assisted ventilation. In India, recurrently reported contributors included TORCH infection, NICU stay, low birth weight, prematurity, ototoxic exposure, craniofacial anomalies, and, in some studies, genetic factors such as connexin 26. Intervention reporting was uneven. Chinese program-level papers emphasized diagnostic completion and system performance more than rehabilitation detail, whereas several Indian hospital studies explicitly referred infants for hearing rehabilitation, including cochlear-implant consideration in some cohorts. Overall, the evidence base still underreports post-diagnostic intervention outcomes in both countries.

### Limitations of the Study

This scoping review presents several limitations that should be acknowledged.

First, as in previous scoping reviews, some relevant data may not have been captured due to the exclusion of non-indexed or non-English publications, particularly in large and heterogeneous countries such as China and India, where substantial information may exist in local reports or institutional databases. Consequently, the available evidence may not fully reflect the actual extent of neonatal hearing screening activities.

Second, most included studies generally provide limited information regarding post-diagnostic intervention strategies. In most cases, once hearing loss is identified, details on rehabilitation pathways, long-term follow-up, or treatment outcomes are either briefly mentioned or not reported. This limitation has been consistently observed in previous European and African scoping reviews and highlights a broader gap in the literature.

Third, the heterogeneity of study designs, screening protocols, and outcome measures across the included studies limits comparability across studies and precludes quantitative synthesis. Variations in screening timing, referral criteria, and follow-up completion rates may significantly influence reported prevalence and coverage estimates.

Finally, the predominance of hospital-based studies, particularly in India, may introduce selection bias and limit the generalizability of findings to the broader population, especially in rural or underserved areas.

## 5. Conclusions

This updated scoping review highlights substantial progress in neonatal hearing screening across Asia, with China and India representing two distinct developmental trajectories. China demonstrates a transition toward large-scale, system-integrated UNHS programs with high and stable coverage, whereas India continues to rely predominantly on hospital-based initiatives with variable implementation and follow-up. Despite this heterogeneity, prevalence estimates in both countries remain broadly consistent with international data, although variability in Indian studies reflects methodological and infrastructural differences. OAE-based screening protocols constitute the backbone of UNHS in both settings, with combined OAE-ABR approaches improving diagnostic accuracy and reducing false positives. However, follow-up adherence and access to confirmatory diagnostics remain critical challenges, particularly in resource-limited and rural contexts. Risk factors for hearing loss are consistent with global evidence, yet post-diagnostic intervention pathways are insufficiently reported, limiting evaluation of long-term outcomes. Overall, while the evidence supports the feasibility and effectiveness of UNHS programs, it underscores the need for stronger integration of screening, diagnosis, and rehabilitation services. Effective integration of these components into national health systems is crucial to reducing the global burden of childhood hearing loss. Future efforts should prioritize stronger integration of screening, diagnosis, and rehabilitation, alongside improved follow-up systems, particularly in resource-limited settings. Standardized protocols and reporting are needed to enhance comparability, while greater focus on long-term outcomes and combined screening approaches may further improve early detection and care delivery.

## Figures and Tables

**Table 1:** The 19 eligible papers after the filtering process. The data are presented in alphabetical order of each state. Abbreviations: NR: Not Reported Information; NSHL: non-syndromic sensorineural hearing loss.

n	Country	Region/City or Town	Sample Size	Study Period	First Author	Year
1	China	Multicenter	14,913	2009–2010	Wang	2011
2	China	Multicenter (8 provinces)	NR	2007–2009	Huang	2012
3	China	Tianjin	58,397	2010–2012	Zhang	2013
4	China	Multicenter	Patients with NSHL NR	NR	Dai	2015
5	China	Southern China	9317	2013–2014	Peng	2016
6	China	Weifang	666	2018–2019	Zhou	2021
7	China	Beijing	1839	2011–2019	Li	2023
8	China	Hospital-based	8631	2017–2024	Ren	2025
9	China	Liuzhou (Guangxi, rural vs. urban)	19,098	2012–2014	Wu	2017
10	China	Shanghai	1,574,380	2002–2012	Chen	2017
11	India	Vellore	500	2006–2007	John	2009
12	India	Chandigarh	2659	2005–2007	Bansal	2008
13	India	Jabalpur	2254	2015–2016	Sachdeva	2017
14	India	Ballabgarh	1720	2011–2014	Dar	2017
15	India	Maval, Pune District (rural Maharashtra)	8192	2014–2016	Parab	2018
16	India	Lucknow	2676	2018–2020	Upadhyay	2022
17	India	Panchkula, Haryana (North)	506	2021–2022	Rawat	2023
18	India	Pune (tertiary care hospital)	5542	2008–2018	Kapadia	2011
19	India	Odisha (policy/program setting)	NR	NR	Sahoo	2024

## Data Availability

No new data were created.

## Supplementary Materials

The following supporting information can be downloaded at: https://www.mdpi.com/article/10.3390/children13060751/s1, Table S1: PRISMA 2020 Checklist [31].

## Abbreviations

AABR	Automated Auditory Brainstem Response
ABR	Auditory Brainstem Response
ASSR	Auditory Steady State Response
BD	bilateral deafness
CHL	Conductive Hearing Loss
CI	Cochlear Implant
DPOAE	Distortion Product OAE
EDHI	Early Hearing Detection and Intervention
ENT	Ear Nose and Throat
HL	Hearing Loss
LBW	Low Birth Weight
LTF	Loss To Follow-up
MHL	Mixed Hearing Loss
NHS	Neonatal Hearing Screening
NICU	neonatal intensive care unit
OAE	Otoacoustic Emissions
PTA	Pure Tone Average
SNHL	Sensorineural Hearing Loss
TEOAE	Transient Evoked OAE
UNHS	Universal Neonatal Hearing Screening

## Appendix A

**Table A1:** Data from China and India considered in the Review.

ID	Country	Population	Studies Selected	Region
1	China	1,416,096,094	10	East Asia
2	India	1,463,865,525	9	South Asia
Selected	2	2,879,961,619	16	1S; 1E

We selected 19 papers from China and India, focusing our investigation on approximately 43% (≈1,253,753,247) of the total Asian population (≈1,924,127,566).
